# Activation of extracellular signal-regulated protein kinase 5 is essential for cystitis- and nerve growth factor-induced calcitonin gene-related peptide expression in sensory neurons

**DOI:** 10.1186/1744-8069-8-48

**Published:** 2012-06-28

**Authors:** Sharon J Yu, Chun-mei Xia, Jarren C Kay, Li-Ya Qiao

**Affiliations:** 1 Department of Physiology and Biophysics, Virginia Commonwealth University School of Medicine, Richmond, Virginia, USA

**Keywords:** ERK5, Akt, NGF, CGRP, DRG

## Abstract

**Background:**

Cystitis causes considerable neuronal plasticity in the primary afferent pathways. The molecular mechanism and signal transduction underlying cross talk between the inflamed urinary bladder and sensory sensitization has not been investigated.

**Results:**

In a rat cystitis model induced by cyclophosphamide (CYP) for 48 h, the mRNA and protein levels of the excitatory neurotransmitter calcitonin gene-related peptide (CGRP) are increased in the L6 dorsal root ganglia (DRG) in response to bladder inflammation. Cystitis-induced CGRP expression in L6 DRG is triggered by endogenous nerve growth factor (NGF) because neutralization of NGF with a specific NGF antibody reverses CGRP up-regulation during cystitis. CGRP expression in the L6 DRG neurons is also enhanced by retrograde NGF signaling when NGF is applied to the nerve terminals of the ganglion-nerve two-compartmented preparation. Characterization of the signaling pathways in cystitis- or NGF-induced CGRP expression reveals that the activation (phosphorylation) of extracellular signal-regulated protein kinase (ERK)5 but not Akt is involved. In L6 DRG during cystitis, CGRP is co-localized with phospho-ERK5 but not phospho-Akt. NGF-evoked CGRP up-regulation is also blocked by inhibition of the MEK/ERK pathway with specific MEK inhibitors U0126 and PD98059, but not by inhibition of the PI3K/Akt pathway with inhibitor LY294002. Further examination shows that cystitis-induced cAMP-responsive element binding protein (CREB) activity is expressed in CGRP bladder afferent neurons and is co-localized with phospho-ERK5 but not phospho-Akt. Blockade of NGF action in vivo reduces the number of DRG neurons co-expressing CGRP and phospho-CREB, and reverses cystitis-induced increases in micturition frequency.

**Conclusions:**

A specific pathway involving NGF-ERK5-CREB axis plays an essential role in cystitis-induced sensory activation.

## Introduction

Cystitis induces considerable changes in the primary afferent pathways that play a significant role in bladder hyperactivity. The molecular mechanism and signal transduction that mediate the cross talk between the inflamed urinary bladder and sensory sensitization has not been investigated. The neuropeptide calcitonin gene-related peptide (CGRP) is enriched in the primary afferent neurons in the dorsal root ganglia (DRG) and is one of the most important nociceptive markers in the control of pain and inflammation [1-10]. Mice lacking CGRP or receiving pharmacological inhibition of CGRP activity do not develop hyperalgesia or central neuropathic pain after inflammation [4-10]. Conversely, mice receiving intrathecal CGRP peptide exhibit nociceptive behavior [11-13]. The involvement of CGRP in nociceptive transmission following noxious stimulation of the peripheral/visceral organ/tissue includes its up-regulation in the DRG [3,5,14-21] and its release centrally to the dorsal horn of the spinal cord [11,16,22,23]. This is also particularly true with cystitis that a previous study by Vizzard [21] shows that chronic irritation of the urinary bladder following multi-dose cyclophosphamide (CYP) treatment causes a CGRP increase in bladder afferent neurons. Thus investigation of the endogenous molecular pathways by which CGRP is regulated in sensory neurons during cystitis will provide insights into the mechanisms underlying visceral inflammation and pain.

In adult rat DRG, about half of the primary sensory populations are peptidergic that are marked by CGRP [24,25]. These cells express the active form of TrkA [26] thus they are able to respond to nerve growth factor (NGF). The action of NGF on CGRP expression in sensory neurons is demonstrated in several forms. In DRG neuronal mass culture, application of NGF increases CGRP transcription [27] in a ras- dependent manner [28]. In animals, intrathecal infusion of NGF can counteract the decrease of CGRP mRNA caused by sciatic nerve transection [29]. In an analogous manner, treatment with NGF antiserum reduces the endogenous level of CGRP in sensory neurons [30] and also prevents the increase in CGRP content in the sciatic nerve of the inflamed paw [31]. In addition to the local action of NGF on CGRP expression, NGF is able to facilitate a retrograde signal by which NGF applied to the extremity of capsaicin-treated rats can counteract capsaicin-induced reduction in CGRP mRNA level in the DRG [32]. These in vitro and in vivo studies suggest a close interrelationship between NGF and CGRP in sensory neurons; however, the detailed signaling transduction pathways that mediate NGF-induced CGRP expression in sensory neurons in animals with disease have yet to be determined.

Three major signaling pathways are activated by NGF binding to TrkA in neurons: the extracellular signal-regulated protein kinase (ERK) pathway, the phosphatidylinositol 3-kinase (PI3K)/Akt pathway, and the phospholipase C (PLC)γ pathway [33]. Activation of ERK (i.e. ERK1/2, ERK5) or PI3K/Akt pathway enhances gene expression through the activation of transcription factor CREB, the cAMP-responsive element binding protein [33-35]. Activation of the PLCγ pathway leads to Ca^2+^ and Na^+^ influx through the activation of ion channels, Ca^2+^ release from stores, and further leads to CREB activation [36]. Considering that the CGRP promoter contains a cAMP-responsive element and CGRP expression is regulated by CRE-mediated transcription [37-39], it is likely that one or more of these pathways can be involved in NGF-induced CGRP expression. A recent study shows that inhibition of mitogen activated protein kinase kinase (MAPKK, MEK) activity blocks the ability of NGF to increase CGRP expression in cultured DRG neurons [28,]. The interplay of the PI3K/Akt pathway in NGF-induced MAPK activation has also been discussed (see review: [40]). In regard to the unique feature of NGF retrograde signaling, activation of MEK/ERK and PI3K/Akt are involved in a region-dependent, isoform-specific manner [41-43]. In sensory neurons, ERK5 rather than ERK1/2 is activated to mediate a retrograde survival response to NGF [34].

Several animal models have demonstrated an elevation of NGF in the inflamed peripheral organs/tissues including hind paw [44], the urinary bladder [45,46], and the distal colon [47]. This target-derived NGF can influence sensory activity via retrograde transport [47]. Previous studies by us [48,49] and others [50] have demonstrated that during cystitis the ERK5 and CREB are activated in bladder afferent neurons and intrathecal application of PD98059, an inhibitor that prevents both ERK1/2 and ERK5 activities [51], significantly decreases micturition frequency in inflamed animals but has no effect on bladder reflex contractions of non-inflamed bladder. Along with this line of research, the present study examines 1) whether endogenous NGF has a role in CGRP expression in the DRG and in inducing bladder overactivity caused by cystitis; 2) whether cystitis-induced CGRP involves NGF retrograde signaling that involves activation of ERK5 and Akt; and 3) the involvement of CREB in NGF signaling. Our results suggest a unique pathway involving ERK5-CREB but not Akt in CGRP up-regulation in the DRG during cystitis.

## Materials and methods

### Experimental animals and reagents

Adult male rats (150–200 g) from Harlan Sprague Dawley, Inc. (Indianapolis, IN) were used. All experimental protocols involving animal use were approved by the Institutional Animal Care and Use Committee at the Virginia Commonwealth University (IACUC # AM10315). Animal care was in accordance with the Association for Assessment and Accreditation of Laboratory Animal Care (AAALAC) and National Institutes of Health guidelines. All efforts were made to minimize the potential for animal pain, stress or distress as well as to reduce the number of animals used. CYP and other chemicals used in this experiment were purchased from Sigma-Aldrich (St. Louis, MO).

### Cyclophosphamide-induced cystitis

CYP cystitis was induced in rats by the technique previously described [52]. Briefly, cystitis was induced in rats by injecting CYP intraperitoneally at a single dose of 150 mg/kg for 48 hours (h). Control rats received volume-matched injections of saline. All injections were performed under isoflurane (2%) anesthesia.

### Anti-NGF and control IgG treatment

A NGF antibody or control IgG (Santa Cruz Biotechnology, Inc. Santa Cruz, CA) was injected intraperitoneally at a dose of 30 μg/kg body weight according to previously published protocol [52]. A single dose of NGF antibody or control IgG was made immediately after the CYP injection. This treatment regimen effectively blocked the action of NGF in the inflamed urinary bladder [52].

### Retrograde labeling

Under anesthesia (2.5% isoflurane, SurgiVet, Smiths Medical PM, Inc. Waukesha, WI), the rat urinary bladder was exposed under a sterile environment with a lower abdominal incision. Neuronal tracing agent Fast Blue (FB, 4%, weight/volume; Polysciences, Inc. Warrington, PA) was injected into 8 sites (5 μL per site) in the bladder wall for retrograde labeling of bladder afferent neurons in the DRG. To prevent leakage and labeling of adjacent tissues, the needle was left in place for 30 sec after each injection and a cotton swab was held close to the injection site to wipe off any excess dye that might leak from the needle tip during the needle withdrawal. In this manner, no visible leakage of the dyes was observed after each injection. Injections into the lumen, major blood vessels, or overlying fascial layers were avoided. The incision was closed with 4–0 sutures. The rats were allowed for survival until the harvest of the tissues.

### Tissue harvesting

For immunohistochemistry, animals were deeply anesthetized with isoflurane (2–3%) and then underwent euthanasia via intracardiac perfusion with oxygenated Krebs buffer (pH 7.4; 95% O2, 5% CO2) followed by 4% paraformaldehyde. The L6 DRGs were identified and sectioned parasagitally at a thickness of 20 μm. For ganglion-nerve preparation, animals were sacrificed with overdose of isoflurane followed by thoracotomy. The L6 DRG along with the distal spinal nerve were freshly dissected out and placed into Dulbecco's Modified Eagle Medium (DMEM) with or without inhibitors for culture. For real-time PCR, the L6 DRG was freshly dissected out and subjected to RNA extraction.

### Immunohistochemistry

An on-slide technique was used for immunostaining of the DRG sections. DRG sections were incubated with blocking solution containing 3% normal donkey serum (Jackson ImmunoResearch, West Grove, PA) in PBST (0.3% Triton X-100 in 0.1 M PBS, pH 7.4) for 30 min, followed by specific primary antibodies overnight at 4°C. These antibodies included mouse anti-CGRP (1:2000, Abcam, Cambridge, MA), rabbit anti-CGRP (1:1000, Millipore, Lake Placid, NY), rabbit anti-phospho-ERK5 (1:400, Cell Signaling Technology, Inc. Danvers, MA), goat anti-phospho-ERK5 (1:200, Santa Cruz Biotechnology, Inc. Santa Cruz, CA), rabbit anti-phospho-Akt (1;500, Cell Signaling Technology, Inc. Danvers, MA), mouse anti-phospho-Akt (1:2000, Cell Signaling Technology, Inc. Danvers, MA), and rabbit anti-phospho (p)-CREB (1:1000, Cell Signaling Technology, Inc. Danvers, MA). After rinsing (3 × 10 min with 0.1 M PBS), tissues were incubated with fluorescence-conjugated species-specific secondary antibody Alexa 594 or 488 (1:500, Molecular Probes, Eugene, OR) for 2 h at room temperature. Following washing, the slides were coverslipped with Citifluor (Citifluor Ltd., London).

DRG cells with visible nucleus were counted with a Zeiss fluorescent photomicroscope. CGRP and p-CREB cell profiles were counted in 6 to 10 sections randomly chosen from each L6 DRG. The area of section containing cells (excluding the area containing fibers) was selected using free-line tools integrated with the AxioVision measurement software (Carl Zeiss, Inc.) and was measured as mm^2^. The number of positively stained cells was normalized against the measured area and expressed as number cells per mm^2^. To avoid double counting, we have chosen every third section for one specific antibody stained.

### RNA extraction and quantitative real-time PCR

Total RNA was extracted using a RNA extraction kit RNAqueous (Ambion, TX). RNA concentration was determined spectrophotometrically. cDNA was synthesized using Cloned AMV First-Strand Synthesis Kit (Invitrogen Life Technologies, Grand Island, NY) with random hexamers. Following reverse transcription, quantitative real-time PCR was performed for CGRP with Taqman probes mixed with PCR Master-Mix for 40 cycles (95 °C for 15 sec, 60°C for 1 min) on a 7300 real-time PCR system (Applied Biosystems, Foster City, CA). Quantitative real-time PCR of the same sample was performed for β-actin expression as internal control. The levels of CGRP mRNA were normalized against β-actin expression in the same sample that was calculated with ΔCt method. The expression levels of the target gene in control animal from each independent experiment was considered as 1, and the relative expression level of these genes in experimental animals was adjusted as a ratio to its control in each independent experiment and expressed as fold changes (2^-ΔΔCt^-fold).

### Examination of voiding behavior

Adapted from a published method for mouse [53], voiding behavior of the rat was analyzed via a non-invasive procedure in which the urine was collected naturally onto an underneath filter paper placed 20 cm below a meshed cage containing the tested animal. We used a cage with a dimension of 25 × 15 × 15 cm^3^ (L × W × H). The number of urine drops from each animal in a 2-h window was counted. Animals treated with CYP excreted more times (i.e., higher number of urine drops) with less volume per drop (i.e., smaller urine spots).

### Statistical analysis

Comparison between control and experimental group was made by using Student's *t*-test. Results were presented as mean ± S.E.M. Differences between means at a level of p ≤ 0.05 were considered to be significant.

## Results

### Cystitis-induced CGRP mRNA and protein levels in the L6 DRG was blocked by inhibition of NGF action in vivo

**Figure 1:**
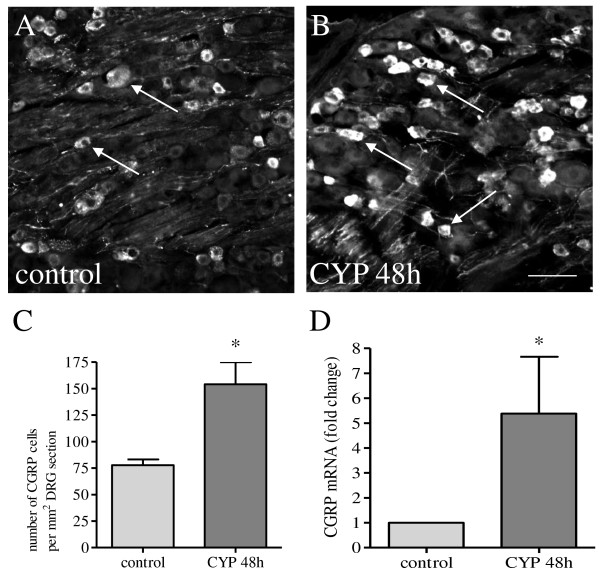
**Cystitis increased CGRP mRNA and protein levels in L6 DRG.** After CYP treatment for 48 h, the CGRP expression was examined in L6 DRG by immunohistochemistry (A-C) and real-time PCR (D). CGRP was largely expressed in small diameter sensory neurons (A, B). The average number of DRG neurons per unit area expressing CGRP was significantly increased post CYP treatment (C). The relative level of CGRP mRNA was also increased in L6 DRG during cystitis (D). Bar = 80 μm. *, p < 0.05 vs control. n = 5-6 animals for each group.

Previous studies have demonstrated that chronic cystitis following multi-dose ten-day treatment with CYP resulted in a significant increase in CGRP immunoreactivity in bladder afferent neurons located in the L6-S1 DRGs [21]. The present study showed that CGRP production was also increased in L6 DRG at 48 h post cystitis induction (Figure 1). Consistently, CGRP immunoreactivity was expressed in small diameter nociceptive neurons (Figure 1A-B). The number of CGRP immunoreactive neurons was significantly increased (p < 0.05) in L6 DRG at 48 h following CYP treatment (Figure 1C). Real-time PCR results showed that CGRP transcript was also elevated in L6 DRG during cystitis (Figure. 1D), suggesting that CGRP was generated by these DRG neurons upon inflammatory irritation of the urinary bladder. 

**Figure 2:**
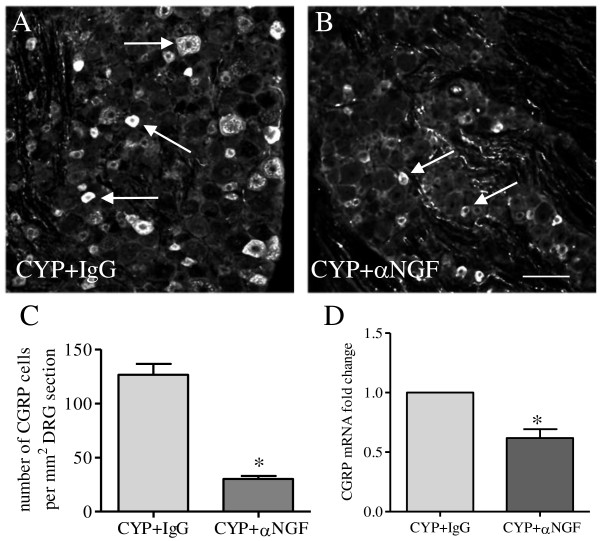
**NGF immuno-neutralization attenuated cystitis-induced CGRP expression in L6 DRG.** The number of CGRP immunoreactive neurons was significantly higher in L6 DRG from animals treated with CYP and control IgG (A, C) when compared to those from animals treated with NGF neutralizing antibody and CYP (B, C). Blockade of NGF activity in cystitis animals also reduced CGRP transcription in the L6 DRG (D). *, p < 0.05 vs CYP + IgG. n = 5 animals for each group. Bar = 60 μm.

It has been well established that NGF serves as an endogenous mediator in some persistent pain states. The CGRP positive peptidergic sensory neurons often express TrkA [26], thus are able to respond to NGF action. To examine whether CGRP up-regulation in the L6 DRG was mediated by endogenous NGF during cystitis, we administered a NGF neutralizing antibody to rats with cystitis to block NGF activity in vivo. Cystitic animals receiving the same amount of control IgG served as comparison. After 48 h post drug treatment, we examined the mRNA and protein levels of CGRP in the L6 DRG (Figure 2). In animals treated with CYP and control IgG, there was an average of 126.6 ± 10.1 CGRP cells per mm^2^ DRG neuronal area (Figure 2A, C). Treatment with NGF neutralizing antibody reduced the number of DRG neurons expressing CGRP to 30.2 ± 2.7 per mm^2^ DRG neuronal area (p < 0.05, Figure 2B, C). Treatment with NGF neutralizing antibody also decreased the CGRP mRNA level in CYP-treated animals when compared to CYP + IgG treatment (Figure 2D), suggesting that endogenous NGF triggered CGRP transcription in the L6 DRG during cystitis. 

### CGRP was co-localized with phospho-ERK5 but not phospho-Akt in L6 DRG during cystitis

**Figure 3:**
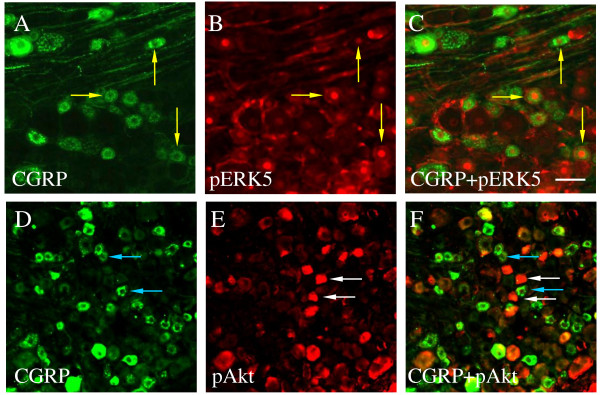
**Co-localization of CGRP with phospho-ERK5 but not phospho-Akt in L6 DRG during cystitis.** Double immunostaining showed that a subpopulation of CGRP cells (A, green cells indicated by yellow arrows) also expressed phospho-ERK5 (B, red nuclear staining). In contrast, CGRP cells did not express phospho-Akt (D-F, blue arrows indicated CGRP cells, white arrows indicated Akt cells).

We have reported that the level of phospho-ERK5 was increased in the DRG during cystitis [48]. ERK5 was also a key molecule activated in the sensory neuronal somata upon NGF retrograde stimulation of cultured DRG neurons [34]. In the present study, double immunostaining of the L6 DRG from animals with cystitis showed that a subpopulation of CGRP cells (Figure 3A, yellow arrows) also expressed phospho-ERK5 (Figure 3B). In contrast, CGRP cells did not express phospho-Akt (Figure 3D-F) even though Akt was also a major downstream intermediate signaling molecule regulated by NGF [33]. These results suggested that activation of ERK5 rather than Akt was likely responsible for CGRP expression in the DRG. 

### Prevention of ERK5 but not Akt activity blocked retrograde NGF-induced CGRP expression in the DRG somata

**Figure 4:**
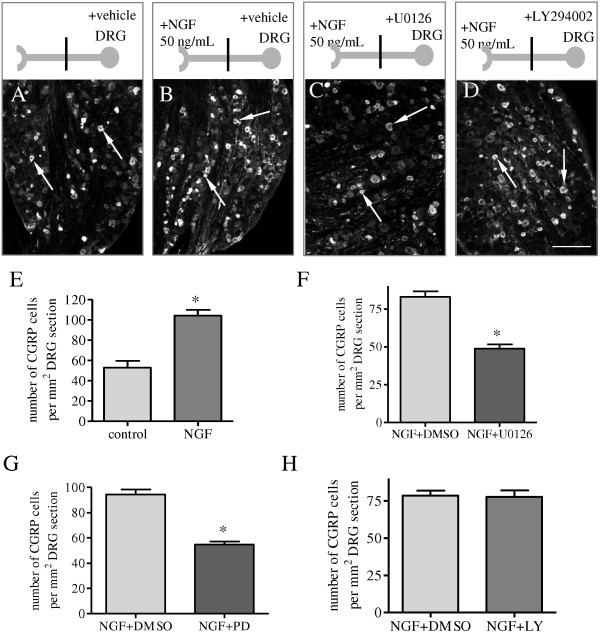
**Retrograde NGF-induced CGRP expression was mediated by the MEK/ERK pathway but not by the PI3K/Akt pathway.** In two-compartmented culture, the gangalia was pre-treated with vehicle (vehicle, A, B, E), or one of the inhibitors against the MEK/ERK pathway (C, F: U0126; G: PD98059), or the PI3K/Akt pathway (D, H: LY294002) followed by NGF treatment of the nerve terminals (B, C, D). After 12 h of NGF treatment, the level of CGRP was increased in the sensory neuronal cell bodies by 2 fold when compared to control (E). Both U0126 (F) and PD98059 (G: PD) pre-treatment blocked the retrograde NGF-facilitated CGRP expression in DRG. LY294002 (LY) pre-treatment did not affect the level of CGRP in the ganglia induced by retrograde NGF (H). Bar = 120 μm. *, p < 0.05 vs vehicle. Results were from 4 independent experiments for each treatment.

Since phospho-ERK5 was co-localized with CGRP in the L6 DRG during cystitis (Figure 3A-C), we then examined whether NGF-induced CGRP in the DRG was mediated by the ERK5 pathway. We utilized a two-compartmented L6 DRG-nerve preparation and examined the effect of retrograde NGF on CGRP expression in the DRG. This system was chosen based on that NGF was elevated in the inflamed urinary bladder [45,46] and its retrograde signal had a critical role in mediating the target tissue-neuron interaction. Our results showed that application of exogenous NGF (50 ng/mL) to the nerve terminals caused a two-fold increase (p < 0.05) in the number of DRG neurons expressing CGRP in the DRG after 12 h of NGF treatment (Compare Figure 4B to 4A, Figure 4E). When we blocked the ERK5 activity with a specific MEK inhibitor U0126 (2 μM) or PD98059 (5 μM), we found that NGF-induced CGRP expression was reduced by these inhibition (Figure 4F, G). In contrast, inhibition of Akt activity with a PI3K inhibitor LY294002 (5 μM) had no effect on NGF-induced CGRP expression in the DRG neurons (Figure 4H). These results suggested that activation of ERK5 but not Akt mediated retrograde NGF-induced CGRP expression in the L6 DRG. 

### CGRP cells co-expressed CREB activity during cystitis

**Figure 5:**
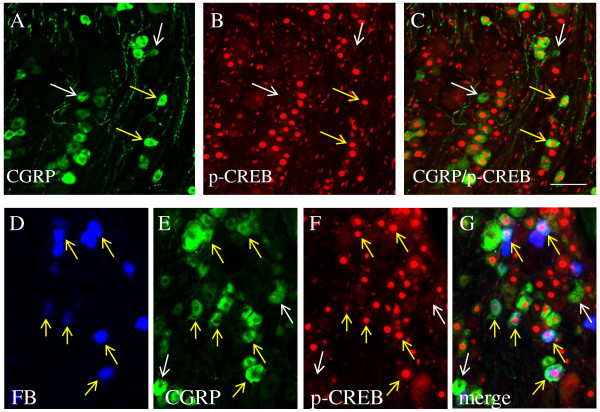
**Co-localization of CGRP with phospho-CREB in bladder afferent neurons during cystitis.** Double immunostaining showed that CGRP cells (A, green cells indicated by yellow arrows) often expressed phospho (p)-CREB (B, red nuclear staining, C was the merged photomicrograph of A and B indicating co-localization of CGRP and p-CREB: yellow arrows) during cystitis. In FB-labeled bladder afferent neurons (D, blue cells indicated by yellow arrows), CGRP (E, green cells) was also co-expressed with p-CREB (F: red nuclear staining; G was the merged photomicrograph of D, E and F indicating bladder afferent neurons expressing both CGRP and p-CREB indicated by yellow arrows). White arrows indicated CGRP neurons that did not have p-CREB. Bar = 60 μm.

**Figure 6:**
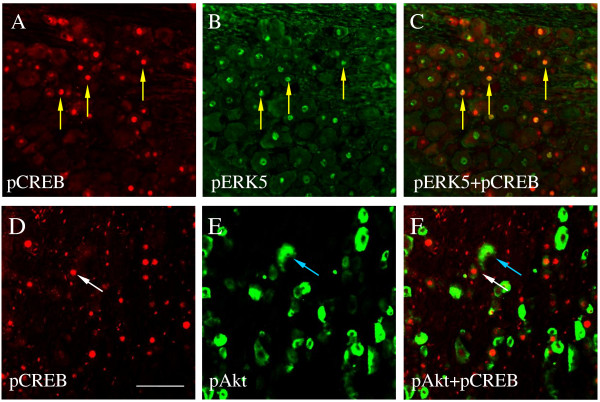
**Co-localization of phospho-CREB with phospho-ERK5 but not phospho-Akt in L6 DRG during cystitis.** Double immunostaining showed that phospho-CREB in L6 DRG during cystitis (A, D: red nuclear staining) was co-localized with phospho-ERK5 (B, C: green nuclear staining) but not phospho-Akt (E, F: green cells). Bar = 60 μm.

The transcription factor CREB was implicated to function as a molecular switch underlying neural plasticity [54]. In cultured sensory neurons, activation of CREB was involved in retrograde NGF-induced sensory neuronal survival response [34]. During cystitis, CREB was also activated in bladder afferent neurons in the L6 DRG [49]. It has been reported that in DRG neuronal culture activation of CREB was a necessary element in NGF-induced CGRP up-regulation [55]. In the present study, we found that during cystitis about 75% CGRP cells (Figure 5A) expressed phospho-CREB (Figure 5B) in the L6 DRG (Figure 5A-C, yellow arrows); CGRP and phospho-CREB were also co-expressed in bladder afferent neurons in the L6 DRG (Figure 5D-G, yellow arrows). It was noteworthy that some of the CGRP neurons did not express phospho-CREB (Figure 5, white arrows). It could be that these CGRP were not caused by cystitis, or CREB in these neurons was deactivated (dephosphorylated) prior to examination. Co-localization studies also showed that phospho-CREB (Figure 6A, D) was co-localized with phospho-ERK5 (Figure 6A-C) but not phospho-Akt (Figure 6D-E) in the L6 DRG during cystitis. 

### Blockade of NGF action in vivo reduced cystitis-induced CREB activation in CGRP neurons and reversed bladder hyperactivity

**Figure 7:**
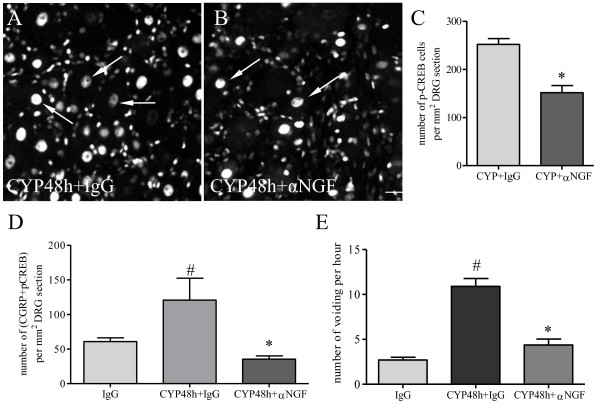
**NGF immuno-neutralization reduced the level of CREB phosphorylation in CGRP neurons during cystitis and reversed cystitis-induced bladder overactivity.** The number of phospho (p)-CREB immunoreactive neurons was significantly higher in L6 DRG from animals treated with CYP and control IgG (A, C) when compared to those from animals treated with NGF neutralizing antibody and CYP (B, C). NGF neutralization also reduced cystitis-induced increases in the number of L6 DRG neurons co-expressing CGRP and p-CREB (D), and reversed cystitis-induced increases in micturition frequency (E). *, p < 0.05 vs CYP + IgG. #, p < 0.05 vs vehicle treatment. n = 4 animals for each group. Bar = 40 μm.

To examine whether NGF induced CREB activation in vivo, we compared the level of phospho-CREB in L6 DRG and in CGRP-expressing neurons in CYP-treated animals receiving either control IgG or anti-NGF treatment. A significant reduction of phospho-CREB was found in L6 DRG in animals treated with anti-NGF (Figure 7B) when compared to control IgG treatment (Figure 7A, C). Cystitis-caused increases in the number of L6 DRG neurons co-expressing CGRP and phospho-CREB were also attenuated by anti-NGF treatment (Figure 7D). Associated with sensory neuronal activation, cystitis significantly increased micturition frequency examined by number of voiding in a 2-h window of recording from unrestraint non-operated conscious animals (Figure 7E), suggesting that these animals exhibited overactive bladder. Anti-NGF treatment reversed cystitis-induced bladder overactivity (Figure 7E).

## Discussion

The key findings of the present study are that activation of the ERK5 but not the Akt pathway is involved in cystitis- and retrograde NGF-induced CGRP expression in primary sensory neurons. A line of evidence shows that the neuropeptides NGF and CGRP have prominent roles in nociceptive transmission and inflammatory pain [1-4,44,56,57]. Viral gene transfer of NGF to the urinary bladder triggers bladder overactivity [57] suggesting the ability of viscerally expressed NGF in regulating sensory activity. However, the molecular pathways by which visceral NGF induces bladder sensory activity is not investigated. In the present study, we combine in vivo and in vitro approaches and demonstrate that NGF regulates sensory activity by activating CREB and CGRP in primary sensory neurons in the DRG, which is mediated by a unique signaling pathway involving activation of ERK5. Following inflammatory irritation of the urinary bladder in animals or patients, the level of NGF is elevated in the viscera [45,46,58,59]. NGF binding to its receptor TrkA may undergo retrograde transport to the DRG where they regulate sensory activity by increasing the ERK5 and CREB activities as well as CGRP production.

**Figure 8:**
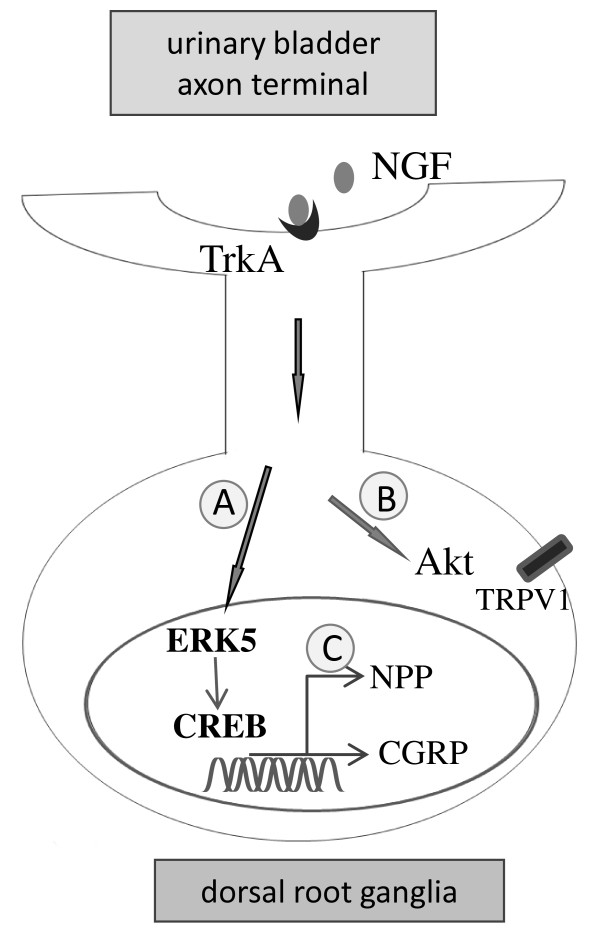
**Schematic diagram illustrates the putative mechanism for NGF signal transduction that mediates the interaction of the inflamed urinary bladder and bladder sensory neurons in the DRG.** Cystitis**-**induced NGF expression in the urinary bladder binds to TrkA and activates ERK5 and CREB in DRG via retrograde transport. The NGF-ERK5-CREB axis leads to CGRP up-regulation in bladder afferent neurons (A). The NGF-induced Akt activation (B) may contribute to TRPV1 sensitization [61] but not the CGRP up-regulation in the DRG. In addition to regulating CGRP expression, activation of CREB in bladder afferent neurons may also control gene expression of other neuropeptides (NPP) (C). These multi-branches of signaling pathways triggered by NGF may together mediate cystitis-induced bladder hyperactivity.

ERK5 is a novel member of the ERK family that is sensitive to cytokine, stress and mitogenic factors. The present study shows that activation of ERK5 (phospho-ERK5) in the L6 DRG during cystitis is associated with CGRP expression and CREB activation. Prevention of ERK activity with a MEK inhibitor PD98059 that blocks both ERK1/2 and ERK5 attenuates retrograde NGF-induced CGRP up-regulation in the DRG neuronal soma. These findings are consistent to published studies in showing that activation of ERK5 is a key pathway in retrograde NGF-induced sensory neuronal survival response [34]. Several studies have also demonstrated that NGF-induced sensitization of the TRPV1 response is attenuated by inhibition of the PI3K/Akt pathway when NGF is applied directly to the neurons [60,61] or injected intradermally [62] suggesting that the PI3K/Akt participates in both local and retrograde NGF action. In our study, prevention of the PI3K/Akt activity fails to block retrograde NGF-induced CGRP expression in the DRG. During cystitis, the phospho-Akt is not co-expressed with either CGRP or phospho-CREB suggesting that the PI3K/Akt pathway is unlikely serving upstream of the pathway leading to CGRP expression and CREB activation in these neurons. Immuno-colocalization study shows that 60% of CGRP DRG neurons contain TRPV1 immunoreactivity [63]; however, there is scarce overlap of TRPV1 and CGRP fibers in the dorsal horn of the spinal cord [63]. These results suggest that PI3K/Akt-mediated TRPV1 and MEK/ERK5-mediated CGRP may have distinct function in mediating sensory activity (Figure 8). 

Cystitis is accompanied with increased urinary urgency, frequency and suprapubic and pelvic pain. Emerging evidence show that inflammatory mediators generated in the urinary bladder triggers bladder sensory activation thereby contributing to bladder hyperactivity [64]. Following CYP treatment, a number of inflammatory mediators are produced and released into the lamina propria where they sensitize the sensory nerve terminals and cause sensory hypersensitivity. The present study along with previous publications demonstrates that NGF is a critical endogenous mediator in cystitis-induced bladder sensory hyperactivity [65]. Blockade of NGF action in vivo not only attenuates cystitis-induced CREB activation and CGRP expression in the DRG but also reverses cystitis-induced increases in micturition frequency. NGF generated in the urinary bladder [45,46] may undergo retrograde transport to regulate gene expression in the DRG. Our study shows that application of NGF to the sensory nerve terminals indeed increases CGRP expression in the DRG neuronal soma. The retrograde NGF action on affecting bladder sensory activity has also been demonstrated by injection of exogenous NGF into the normal rat bladder which results in bladder hyperactivity [57]. The present study provides a molecular basis for the physiological role of NGF in regulating bladder activity which is that NGF in the urinary bladder sensitizes bladder afferent neurons by regulating CRE-mediated gene expression such as CGRP.

The interplay between NGF and CGRP pathways has long been suggested. Injection of NGF (alphaNGF) antiserum to nonoperated animals decreases the levels of CGRP protein expressed in DRG [30]. CGRP mRNA in DRG was also absent from TrkA−/− mice as well as in NGF-deprived DRG explants [66]. In the present study, we demonstrate that injection of NGF antibody reverses both the elevated levels of CGRP mRNA and protein in L6 DRG induced by cystitis. The promoter region of the CGRP gene contains a consensus sequence responsive to the transcription factor CREB [37]. In L6 DRG during cystitis, a large population of CGRP neurons contains phospho-CREB. This suggests that CREB may also be involved in NGF signaling during cystitis. It has been reported that retrograde NGF regulates CREB activation in cultured rat sympathetic neurons, and plays a critical role in neuronal plasticity [54]. Consistent with this notion, our results show that in cystitis endogenous NGF facilitates CREB activation in primary sensory neurons because NGF antibody treatment blocks cystitis-induced CREB activation in L6 DRG. There are also parallel decreases in the CGRP expression along with CREB activation in DRG neurons co-expressing both molecules following NGF antibody treatment of the cystitis animals. Taken together, these results suggest that NGF regulates sensory activity and CGRP expression involves CREB activation during cystitis. CREB can be activated by a number of kinases including the Ca^2+^/CaM-dependent kinase II, PKA, and MAPK and Akt [67], and occupies approximately 4,000 promoter sites in human tissues [68]. Thus, in addition to CGRP, other neuropeptides and ion channels may also be regulated by CREB in sensory neurons (Figure 8). This is shown consistently in our studies that in the L6 DRG during cystitis many phospho-CREB neurons do not express CGRP.

Examination of retrograde pathways that are initiated by NGF leading to CGRP expression in DRG shows that application of specific inhibitors against the MEK/ERK pathway blocks retrograde NGF-induced CGRP up-regulation in the sensory neuronal cell body, while inhibition of the PI3K/Akt pathway has no effect. Up-regulation of CGRP by the ERK MAPK pathway has also been demonstrated in trigeminal ganglia neurons [69]. It is noteworthy that the current study does not preclude the possibility of other factors in regulating CGRP expression in the DRG. These factors include but are not limited to growth factors, cytokines, purinergic system, and glutamate and receptors that are also elevated in the inflamed bladder and/or sensory pathways during cystitis [64]. Cytokine activin is able to increase CGRP expression in sensory neurons in culture and in vivo after peripheral inflammation [70,71]. It is shown that activin acts synergistically with NGF in inducing CGRP expression in sensory neurons [72].

In conclusion, the present study demonstrates that activation of a unique signaling involving activation of ERK5 but not Akt in cystitis- and NGF-induced CGRP expression in the DRG suggests that target of ERK pathway may be a potential therapeutic strategies in treatment of bladder pain with cystitis.

## Competing interest

The authors declare that they have no competing interests.

## Authors’ contributions

SJY, LYQ designed and conducted most of the experiments. CMX, JCK conducted some of the experiments. SJY, LYQ wrote the manuscript. All authors analyzed the data, read and approved the final version of the manuscript.

## Supporting Grant

NIH DK077917 (LYQ)
